# Image-Guided Internal Fixation of an Oblique Sagittal Split Fracture of C1 Lateral Mass with Motion Preservation: A Technical Report

**DOI:** 10.7759/cureus.1496

**Published:** 2017-07-20

**Authors:** James G Malcolm, Lee A Tan, Andrew K Johnson

**Affiliations:** 1 Neurosurgery, Emory University; 2 The Spine Hospital, Columbia University Medical Center; 3 Neurosurgery, Swedish Covenant Hospital, Chicago, Il

**Keywords:** cervical spine trauma, motion preservation, image-guided, cervical spine fractures, spinal fixation, c1 sagittal split fracture, intraoperative navigation

## Abstract

A sagittal split fracture of the C1 lateral mass is an unstable subtype of C1 fractures and has a high propensity for developing late deformities and pain with nonoperative management. A primary internal fixation of this type of fracture has been recently described with good clinical outcomes and preservation of motion. We present a modified technique of primary internal fixation using an obliquely inserted C1 lag screw with imaging guidance. We successfully treated a 55-year-old woman with a unilateral C1 oblique sagittal split fracture who failed nonoperative management. Technical nuances are discussed with a review of pertinent literature.

## Introduction

Unilateral C1 lateral mass sagittal split fractures are unstable subtypes of C1 fractures that can often lead to a cock-robin deformity, where the head is rotated toward and tilted away from the affected side with significant pain and restricted motion [1]. A displacement of the fractured C1 lateral mass by the occipital condyle causes the settling of the occipital condyle on the C2 lateral mass [1-2]. Primary internal fixation using bilateral C1 lateral mass screws connected by a rod has been shown to yield a good clinical outcome with motion preservation [2]. However, when the fracture line has an oblique orientation, a standard C1 lateral mass screw trajectory does not offer bony purchase into the anterior fragment. We describe a modified C1 primary fixation technique using imaging guidance to insert an obliquely oriented C1 lateral mass screw for the reduction and fixation of a C1 lateral mass split fracture.

## Technical report

Presentation

**Figure 1:**
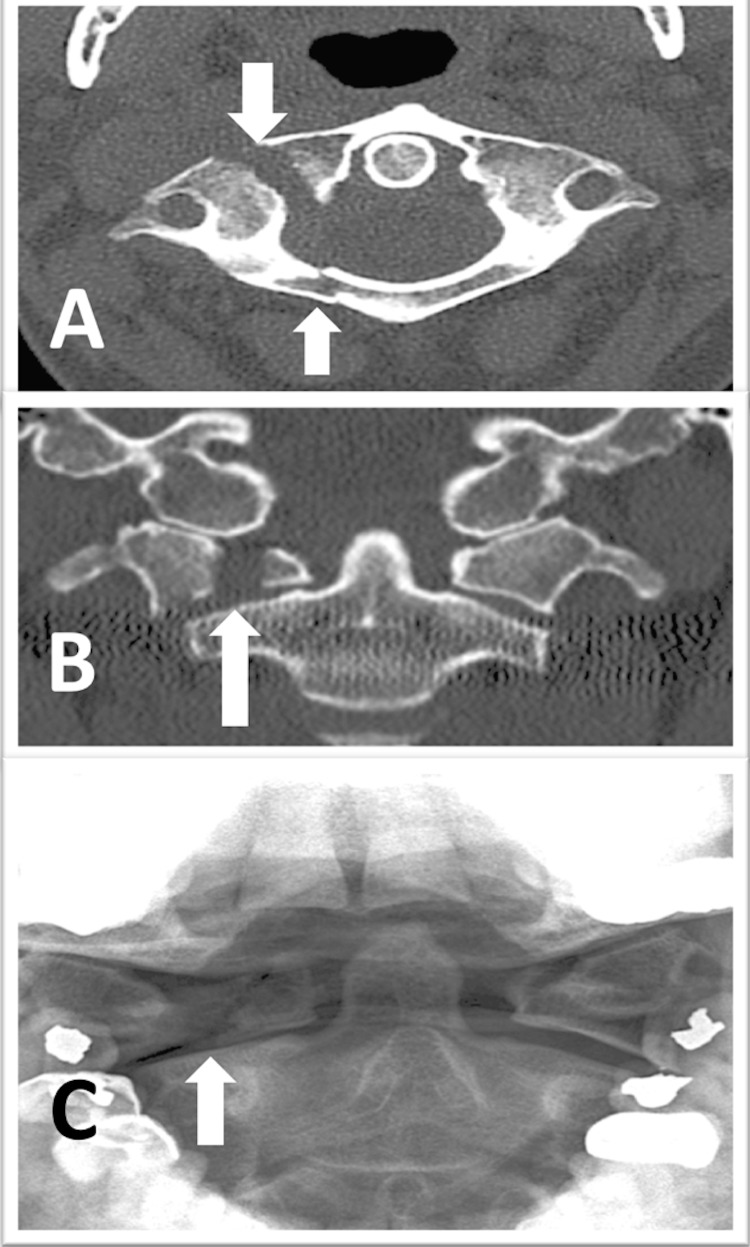
Preoperative images Preoperative images demonstrating the C1 oblique right split fracture (arrows): (A) an axial computed tomography (CT) scan showing an oblique fracture line through the lateral mass and the posterior arch; (B) a coronal CT scan showing a 5 mm overhang of C1 over C2; (C) an open-mouth odontoid x-ray after two weeks of rigid orthosis showing the settling of the C1 right lateral mass

A 55-year-old woman was found to have a right C1 lateral mass sagittal split fracture and a C1 posterior ring fracture after a fall. The fracture line passed obliquely through the right lateral mass of C1 (Figure 1A), and there was a 5 mm overhang of C1 over the C2 lateral mass (Figure 1B). Magnetic resonance imaging (MRI) did not suggest transverse atlantal ligament (TAL) disruption and the examination was unremarkable. After two weeks in rigid orthosis, she presented with progressive pain, the cock-robin deformity, and bony settling (Figure 1C).

Surgical treatment

Axial traction was applied for two days preoperatively. In the operating room, the patient was placed in the prone position with a radiolucent Mayfield head clamp and continued axial traction. A standard midline incision was made from the inion to below the C2 spinous process and the suboccipital skull surface, the C1 posterior arch, and the C2 lateral masses were exposed in the usual fashion. The reference frame from image guidance (Stealth, Medtronic, Memphis, USA) was attached to the C1 posterior arch opposite the fracture and the cervical traction was removed.

**Figure 2:**
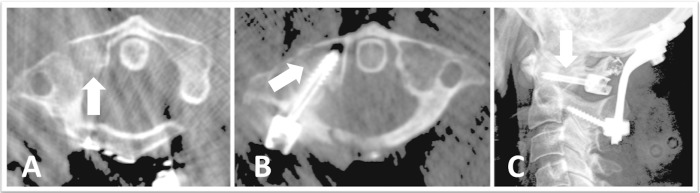
Intraoperative CT and postoperative x-ray after stage 1 An intraoperative axial computed tomography (CT) scan demonstrating the placement of a lag screw during the first stage and showing (A) the fracture line; (B) the lag screw passing through the lateral mass with fracture reduction; and (C) a postoperative x-ray demonstrating the occipitocervical instrumentation after the first stage of the procedure

Using O-arm imaging guidance (Medtronics, Dublin, Republic of Ireland), a screw trajectory was planned in a posterolateral to anteromedial direction, almost perpendicular to the fracture line. This trajectory entered the lateral mass posterior to the vertebral artery and the transverse foramen passing across the fracture line into the anterior arch (Figures 2A and 2B). To insert the screw, a stab skin incision was made and the underlying superficial cervical fascia was opened with cautery. The drill guide was pushed gently to pierce the deep cervical fascia overlying the C1 arch. To avoid force directed toward the spinal cord and vertebral artery, the skin and muscle tissue were rotated outward and the deep fascia was punctured with the instrument pointed in a coronal trajectory behind the C1 arch.

To compensate for the anticipated reduction, the drill and the drill guide were angled slightly anterior to the planned trajectory. After the fractured fragment was partially drilled, gentle pressure was applied to the drill guide to further reduce the fracture and to align its trajectory into the anterior arch before drilling into the anterior arch. Since the anterior arch was connected to the navigation clamp, image guidance remained accurate for this final part of the drilling. A Kirschner wire (K wire) was placed through the drill guide to maintain position. A 4.0x28 mm lag screw with a 10 mm threaded tip and a tulip was passed over the K wire to reduce and fix the fracture (Figure 2B). Internal fixation was achieved using standard occiput C2 instrumentation while skipping C1 and minimizing arthrodesis to prevent segmental fusion (Figure 2C).

After 18 weeks, the patient was brought back for the removal of the occipitocervical instrumentation and the tightening of the lag screw. To provide stronger permanent fixation, a 4.0x32 mm C1 lateral mass screw was placed into the contralateral C1 lateral mass and connected to the primary lag screw with a 60 mm rod. The patient tolerated the procedure well without any complications.

**Figure 3:**
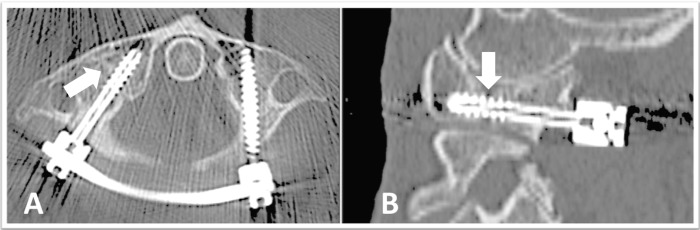
Final postoperative images A follow-up computed tomography (CT) scan at six months after the second stage with (A) axial and (B) sagittal views demonstrating a stable C1 screw-rod construct with evidence of bone growth across the fracture

A postoperative CT scan at six months demonstrated a stable C1 instrumentation with evidence of bone healing across the fracture line (Figure 3). Fifteen months after her second-stage procedure, she had a full range of motion in the cervical spine, no neurologic deficit, and minimal pain.

## Discussion

The craniovertebral junction is a highly mobile region responsible for a majority of the head and neck motion. The atlanto-occipital joint allows a large degree of flexion and extension, while the atlantoaxial joint allows a significant amount of axial rotation.

Isolated C1 fractures with intact TAL are generally considered stable and treated conservatively, while C1 fractures with ruptured TAL are usually managed surgically [3]. Bransford et al. reported that a high percentage of patients with C1 lateral mass sagittal split fractures with intact TAL can still develop pain and cervical deformity as the occipital condyle displaces the fractured C1 lateral mass and settles on the C2 lateral mass [1-2]. The authors initially treated these fractures using occopitocervical fixation, but later transitioned to using the primary internal fixation of C1 to preserve motion [1-2]. This primary fixation technique was originally described by Bohm et al. to treat unstable Jefferson fractures where bilateral C1 lateral mass screws were crosslinked by a rod [4]. Bransford et al. demonstrated the effectiveness of this technique in three patients with C1 lateral mass sagittal split fractures [2].

Our patient had a displaced sagittal split fracture of C1 with an obliquely oriented fracture line. To reduce the displaced anterior lateral mass fragment, we used an obliquely inserted lag screw. Given the neurovascular anatomy in this region, imaging guidance was utilized to facilitate the safe and successful placement of the screw.

The advantages of this lateral screw trajectory include 1) the ability to treat variant pathologies, 2) avoidance of the region posterior to the lateral mass, which often requires tedious dissection or transection of the C2 nerve, 3) additional bony purchase including the anterior arch, and 4) avoidance of a trajectory aimed at the hypoglossal nerve. The disadvantages of this approach include 1) the need for an additional incision and soft tissue dilation, 2) the need for navigation and its potential inaccuracies, and 3) unfamiliar trajectories making fluoroscopy difficult.

Several lessons can be learned from our initial experience. First, we question whether the occipitocervical fixation provided the necessary support. A single stage C1 screw-rod construct similar to the final construct may have been sufficient and avoided the second stage of surgery. Second, piercing the cervical fascia with a large dilator required significant force, which can be dangerous and should not be performed with force directed at the spinal cord or vertebral artery. For this maneuver, the myocutaneous flap was externally rotated to allow a safer posterior trajectory.

## Conclusions

The unique anatomy in the craniovertebral junction makes some fracture types difficult to treat. We presented a staged surgical approach to successfully treat an oblique sagittal lateral mass fracture of C1 with motion preservation.
